# Endometrioid ovarian carcinoma landscape: pathological and molecular characterization

**DOI:** 10.1002/1878-0261.13679

**Published:** 2024-06-25

**Authors:** Alexandre de Nonneville, Elsa Kalbacher, Francesco Cannone, Arnaud Guille, José Adelaïde, Pascal Finetti, Maria Cappiello, Eric Lambaudie, Giuseppe Ettore, Emmanuelle Charafe, Emilie Mamessier, Magali Provansal, François Bertucci, Renaud Sabatier

**Affiliations:** ^1^ Department of Medical Oncology Aix‐Marseille Univ, Inserm, CNRS, Institut Paoli‐Calmettes Marseille France; ^2^ Aix‐Marseille Univ, Inserm, CNRS, Institut Paoli‐Calmettes, CRCM—Predictive Oncology Laboratory Marseille France; ^3^ Department of Medical Oncology CHRU Jean Minjoz Besançon France; ^4^ Department of Obstetrics and Gynecology ARNAS Garibaldi Catania Italy; ^5^ Department of Surgical Oncology Aix‐Marseille Univ, Inserm, CNRS, Institut Paoli‐Calmettes Marseille France; ^6^ Department of Biopathology Aix‐Marseille Univ, Inserm, CNRS, Institut Paoli‐Calmettes, ICEP platform, CRCM Marseille France; ^7^ ARCAGY‐GINECO, GINEGEPS Group Paris France

**Keywords:** endometrioid carcinoma, gene expression profiling, genomic, ovarian cancer, tissue microarray

## Abstract

Endometrioid ovarian cancers (EOvC) are usually managed as serous tumors. In this study, we conducted a comprehensive molecular investigation to uncover the distinct biological characteristics of EOvC. This retrospective multicenter study involved patients from three European centers. We collected clinical data and formalin‐fixed paraffin‐embedded (FFPE) samples for analysis at the DNA level using panel‐based next‐generation sequencing and array‐comparative genomic hybridization. Additionally, we examined mRNA expression using NanoString nCounter® and protein expression through tissue microarray. We compared EOvC with other ovarian subtypes and uterine endometrioid tumors. Furthermore, we assessed the impact of molecular alterations on patient outcomes, including progression‐free survival (PFS) and overall survival (OS). Preliminary analysis of clinical data from 668 patients, including 86 (12.9%) EOvC, revealed more favorable prognosis for EOvC compared with serous ovarian carcinoma (5‐year OS of 60% versus 45%; *P* = 0.001) driven by diagnosis at an earlier stage. Immunohistochemistry and copy number alteration (CNA) profiles of 43 cases with clinical data and FFPE samples available indicated that EOvC protein expression and CNA profiles were more similar to endometrioid endometrial tumors than to serous ovarian carcinomas. EOvC exhibited specific alterations, such as lower rates of PTEN loss, mutations in DNA repair genes, and P53 abnormalities. Survival analysis showed that patients with tumors harboring loss of *PTEN* expression had worse outcomes (median PFS 19.6 months vs. not reached; *P* = 0.034). Gene expression profile analysis confirmed that EOvC differed from serous tumors. However, comparison to other rare subtypes of ovarian cancer suggested that the EOvC transcriptomic profile was close to that of ovarian clear cell carcinoma. Downregulation of genes involved in the PI3K pathway and DNA methylation was observed in EOvC. In conclusion, EOvC represents a distinct biological entity and should be regarded as such in the development of specific clinical approaches.

## Graphical abstract

We used tissue microarray, mRNA and DNA analyses to compare endometrioid ovarian cancer (EOvC) with serous ovarian cancer and endometrioid endometrial cancer. We observed that EovC exhibit specific protein, mRNA, and DNA profiles. PTEN expression was prognostic with shorter progression‐free survival in patients with PTEN loss. Our observations highlight the need to develop research programs dedicated to rare subtypes of ovarian cancer.

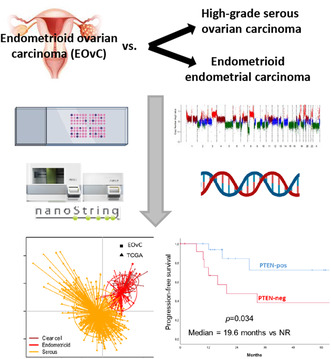

**Abbreviations**
aCGH: array‐comparative genomic hybridization array
CNA: copy number alteration
EOvC: endometrioid ovarian cancer
ER: estrogen receptor
FFPE: formalin‐fixed paraffin‐embedded
GICTIC: Genomic Identification of Significant Targets in Cancer
HRR: homologous recombination repair
IHC: immunohistochemistry
MMRd: mismatch repair deficient
NSMP: non‐specific molecular profile
OS: overall survival
PFS: progression‐free survival
PR: progesterone receptor
SerOvC: serous ovarian carcinomas
TCGA: the Cancer Genome Atlas
TMA: tissue micro‐array

## 1 Introduction

Ovarian cancer is the leading cause of death among gynecological cancer. Every year, 220 000 women will be diagnosed with ovarian cancer worldwide, resulting in 150 000 deaths [1]. The five‐year survival rate is around 45%; however, significant variations exist among patients due to the morphological and molecular heterogeneity of this disease. Epithelial ovarian cancer encompasses four subtypes, with serous carcinoma being the most common, accounting for 75% of all cases. The remaining subtypes, namely mucinous, clear cell, and endometrioid, are less prevalent.

Endometrioid ovarian carcinoma (EOvC) represents approximately 5–10% of epithelial ovarian carcinomas and may be secondary to endometriosis lesion [2]. Median age at diagnosis (~ 53 years) is younger than high‐grade serous carcinoma. EOvC are generally associated with a more favorable prognosis features, such as low grade and early stage at diagnosis [3, 4]. Survival of EOvC is indeed better in EOvC compared with serous ovarian carcinoma (SerOvC). However, these benefits are mainly driven by earlier stage at diagnosis [5, 6].

Around 15% of ovarian carcinomas are linked to germline mutations, particularly alterations in *BRCA1* and *BRCA2* genes. *BRCA1/2* mutations are rare in EOvC, with an incidence of 5–10%, and limited to high‐grade SerOvC [7, 8]. *TP53* mutations are ubiquitous in high‐grade SerOvC and can be identified in 25% of EOvC and are associated with higher genomic instability [9]. The expression of estrogen (ER) and progesterone (PR) receptors is frequent in EOvC, even though low PR expression is correlated to higher grade, genomic instability, and poor survival. Other alterations arising from endometriosis lesions have been described: SWI‐SNF complex (including ARID1A and EZH2) alterations [10, 11, 12, 13] and PIK/AKT pathway (including PTEN loss) dysregulation [14, 15]. Mismatch repair deficient (MMRd) ovarian tumors, associated with Lynch Syndrome, are predominantly endometrioid carcinomas (prevalence of 7–10% in this subtype), whereas MMRd has been rarely reported in other histological subtypes [4, 16, 17]. Limiting testing to EOvC may facilitate the detection of Lynch syndrome in ovarian cancer and select the better candidates for assessing immune checkpoint inhibitors in ovarian cancer [18, 19, 20], as is currently accepted for MMRd endometrial tumors [18, 19, 20, 21, 22, 23]. Moreover, predictive biomarkers of immunotherapy efficacy will need to be identified in this population, and PD‐L1 expression and tumor mutation burden are associated with efficacy in endometrioid endometrial cancer [24].

Previously published EOvC analyses have been focused on specific single approaches (clinical features, protein expression, gene expression analysis, etc.) but no integrated study combining protein, DNA, and mRNA data has been proposed so far. The objective of this retrospective clinical and multi‐omics study is to provide additional insights into the molecular characterization of EOvC. The main goal is to determine the prevalence of major molecular alterations, including P53, PI3K pathway alterations, and mismatch repair deficiency, as well as their prognostic value. These alterations have been investigated using a multimodal approach that combines high‐throughput DNA, mRNA, and protein analyses.

## 2 Materials and methods

### 2.1 Patient characteristics

Our study was retrospective and multicentric. As preliminary clinical analysis, we first retrieved cases recorded in three hospitals specialized in ovarian cancer management (Institut Paoli Calmettes, Marseille, France; CHRU Jean Minjoz, Besançon, France; Garibaldi Nesima Hospital, Catania, Italy). Patients with EOvC (from all three institutions) or SerOvC (all from Institut Paoli‐Calmettes) diagnosed from 2000 to 2016 were included. Other rare subtypes of ovarian carcinoma and patients with non‐ovarian endometrioid tumors were excluded. The endometrioid histology was confirmed centrally by the ICEP (IPC/CRCM experimental pathology) platform (Pr Charafe).

We collected clinical (age and body mass index at diagnosis, hypertension, diabetes, personal and family history of cancer, FIGO stage), treatment (complete macroscopic resection, administration of first‐line chemotherapy, and details of cytotoxic drugs used), and pathological (grade) data from electronic medical files.

The main aim of this translational study was to perform a multi‐omics analysis. We focused our work on cases with FFPE samples available. Considering the sample size, and as EOvC subtype comprises both low‐grade and high‐grade tumors, we did not limit our study to a specific histological grade. High‐grade SerOvC and endometrioid endometrial carcinoma were used as control for TMA analysis. High‐grade SerOvC, endometrioid, serous, and clear cell endometrial carcinoma, as well as normal endometrial cases, were used for gene expression analysis. High‐grade SerOvC, endometrioid, serous, and mixed endometrial carcinoma were used as control for copy number analysis. No low‐grade serous ovarian cancer was used as control.

### 2.2 Immunohistochemistry

IHC methods have been described in earlier papers [25], briefly, for cases with FFPE samples available, we analyzed the expression of 10 proteins known to be frequently altered in ovarian cancer or suspected to be involved in pathways of interest. We assessed the expression of ER (estrogen receptor, EP1 clone – Dako/Agilent, Santa Clara, CA, USA), PR (progesterone receptor, PgR 636 clone – Dako/Agilent), P53 (DO7 – Dako/Agilent), PTEN (6H2.1, Dako/Agilent), PDL‐1 (22C3 pharmDx – Dako Omnis, Santa Clara, CA, USA), mismatch repair proteins [MLH1 (ES05 – Dako/Agilent), PMS2 (EP51 – Dako/Agilent), MHS2 (FE11 – Dako/Agilent), MSH6 (EP49 – Dako/Agilent), and EZH2 (D2C9 – Cell Signaling, Danvers, MA, USA)]. All immunohistochemistry (IHC) experiments were performed using a Tissue MicroArray (TMA) with each case analyzed in duplicate with cores of 1 mm of diameter. All stainings were performed using the Dako Link or Dako Omnis (for PDL1) autostainers (Agilent technologies™) with antibodies used at ready‐to‐use concentration, except for P53, PTEN, and EZH2 for which antibodies were diluted at 1/100, 1/50, and 1/2000, respectively. Antibodies staining were incubated for 20–40 min and revealed with the EnVision Flex kit (Agilent technologies™), according to the manufacturer's instructions (Table S1). Mouse linkers were used for PgR, PTEN, PDL1, MLH1, and MSH2. A rabbit linker was used for PMS2 [26, 27, 28, 29, 30]. The threshold for positivity was set at 10% for hormone receptors expression, 15% for mismatch repair proteins, 10% for PTEN, and diffuse (at least 80%) aberrant nuclear positivity for P53. Cases with no P53 staining, i.e. 0%, were not considered as mutant as we could not rule out that P53 expression may be heterogeneous and missed with limited TMA examination. All tumors with at least 1% of positive cells were classified as EZH2 positive. We used high‐grade serous ovarian tumors (low‐grade serous ovarian tumors were not included) as well as endometrioid uterine tumors as controls to differentiate organ‐related specificities from abnormalities associated with endometrioid histology. Another TMA was developed for these control cases but experiments and analyzes were done concomitantly to that of EOvC tumors.

### 2.3 Array‐comparative genomic hybridization (aCGH)

aCGH methods have been described in earlier papers [25, 31], briefly, DNA was isolated from FFPE blocks or hematoxylin–eosin‐safran slides by automated methods using the EZ‐1 tissue kit, QIAGEN™, according to the manufacturer's instructions.

Array‐CGH was performed to study DNA copy number profiles. After DNA extraction from FFPE blocks or hematoxylin–eosin‐safran slides, cases with DNA of sufficient quality [determined on Agilent Bioanalyzer (Agilent Technologies, Massy, France)] and sufficient amount were analyzed as previously described [32]. Genomic data from the Cancer Genome Atlas (TCGA) ovarian cancer and endometrial cancer datasets were used as controls [33, 34].

### 2.4 Target next‐generation sequencing (t‐NGS)

t‐NGS methods have been described in earlier papers [25], briefly, Panel‐based next‐generation sequencing was conducted in cases with DNA that passed quality controls. For each tumor sample, a library of all coding exons and intron–exon boundaries of a panel of 794 target genes (Table S2) was constructed using the SureSelect enrichment system (Agilent Technologies, Santa Clara, CA, USA). Sequencing was carried out using the Illumina NextSeq500 device (San Diego, CA, USA), according to the manufacturer's instruction at a median depth of 162×.

### 2.5 Transcriptomic analysis

Transcriptomic analysis methods have been described in earlier papers [25], briefly, for cases with sufficient tumor area, RNA was isolated and RNA templates were analyzed using the NanoString nCounter® Dx Analysis System [35]. A dedicated custom gene panel was developed including the main genes implicated in gynecological cancers. This custom panel included 29 genes (Table S3) plus all genes from the Pan‐Cancer pathway panel (770 genes from 13 canonical pathways). Two hundred to 500 ng of total RNA were used as input and sample hybridization was performed according to the manufacturer's instructions. Sample detection and analysis were completed on an nCounter® Digital Analyzer where genes were counted by scanning 555 Fields‐of‐view.

### 2.6 Bioinformatic analyses

Bioinformatic analyses methods have been described in earlier papers [25, 36, 37, 38]. aCGH. All probes for aCGH were mapped according to the hg19/NCBI human genome mapping database. Log2 ratios were segmented with Circular Binary segmentation (CBS) algorithm. We used two different threshold values (log2 ratio > |0.15| and |0.9|) to distinguish low (gain/loss) from high (amplification/deletion) level copy‐number‐alterations (CNA), respectively. The percentage of genome altered was calculated as the sum of altered probes divided by the total number of probes. To identify recurrent copy number alterations, we used the Genomic Identification of Significant Targets in Cancer (GISTIC) 2.0 algorithm calculated by multiple random iterations, with an amplification/deletion threshold > 0.9, confidence level of 0.90, and a corrected threshold probability *q* < 0.25. Genomic signatures exposure was explored according to Macintyre's algorithm [39].

#### 2.6.1 Single nucleotide mutations analyses

Tumor DNA was sequenced using an in‐house panel of genes as previously described [40]. Sequence data were aligned to the human genome (UCSC hg19); alignment and variants calling and annotation were processed as previously described [41].Gene expression profiling (nCounter® platform, Nanostring™, Seattle, WA, USA). Raw data processing, quality control, and normalization were performed using the nSolver™ 4.0 analysis software. Briefly, data processing of raw counts was done with background subtraction defined by the geometric mean of the eight negative control probes. Next, the quality control of samples was checked according to manufacturer requirements in the nSolver™ 4.0. Finally, normalization was done with the geometric mean algorithm using the 40 housekeeping and the six positive control probes. Processed data were then log2‐transformed prior analysis. An unsupervised analysis was done using hierarchical clustering using the Cluster program with data median‐centered on genes [42], Pearson correlation as similarity metrics, and centroid linkage clustering as parameters. Results were displayed using treeview program [42]. In association with hierarchical classification, we used quality Threshold (qT) clustering to select clusters of genes by specifying minimum correlation and size values.

#### 2.6.2 Gene expression profiling of public data

Our Nanostring data were completed by public transcriptomic data sets, i.e., the Uterine Corpus Endometrial Carcinoma and Ovarian Carcinoma data sets from TCGA, following Illumina RNA‐seq processing, normalization, and publication through the UCSC Xena database [43]. We then merged the datasets by using COMBAT (empirical Bayes) as batch effects removal method [44], included in the insilicomerging r/bioconductor package [45]. When multiple probes were mapped to the same GeneID, we retained the one with the highest variance. Uniform Manifold Approximation and Projection (UMAP) as dimension reduction algorithm assessed the normalization of the merged data sets including 786 commons genes. The 410 endometrial endometrioid tumors (out of 560 samples) and the 576 ovarian high‐grade serous tumors from TCGA were selected for further analysis with our 43 EOvCs as well as 66 EOvCs from another set [46]. Eleven normal ovarian samples from two data sets [34, 46] were also used as controls. Supervised analysis was done using a moderated t‐test with empirical Bayes statistic [47] included in the limma r package (version 3.5.2; http://www.cran.r‐project.org/). False discovery rate was applied to correct the multiple testing hypothesis: the significant genes were defined by *P* < 0.05%, *q* < 1%, and fold change (FC) superior to |1.5×| [48]. Using the Gene Expression Signature (GES) obtained each sample was classified using the nearest centroid algorithm. The robustness of the classifier was done by 10‐fold cross‐validation using 1000 iterations using the same parameters as supervised analysis and prediction accuracy was assessed using an exact binomial test with the greater one‐sided hypothesis.

### 2.7 Statistical analyses

Pearson's *χ* ^2^ test (categorical variables) and Wilcoxon test (continuous variables) were used to compare descriptive variables. PFS (progression‐free survival) was defined as the time from diagnosis to disease relapse, progression, or death from any cause. OS (overall survival) was defined as the time from diagnosis to death from any cause. Cause of death was collected to discriminate cancer‐related events to death from other causes. Data concerning patients without disease progression or death at the last follow‐up were censored. Survival curves were estimated using the Kaplan–Meier method. Follow‐up was estimated by the reverse Kaplan–Meier method. The prognostic impact of clinicopathological features was assessed by the Cox regression method in univariate analysis and *P*‐values estimated with the Wald test. All statistical tests were two‐sided at the 5% level of significance. This work was done according to the Strengthening the Reporting of Observational Studies in Epidemiology criteria [49].

### 2.8 Ethics approval and consent to participate

All procedures performed in this study involving human participants were done following the French ethical standards and with the 2008 Helsinki Declaration. Ethical approval was given by the ethics committee of the Institut Paoli‐Calmettes (MEDOC‐IPC 2017‐031). All patients have been informed that their data could be used for further research studies. Dated and signed informed institutional consents have been individually collected for biological analyzes.

## 3 Results

### 3.1 Phenotypic characteristics

**Fig. 1:**
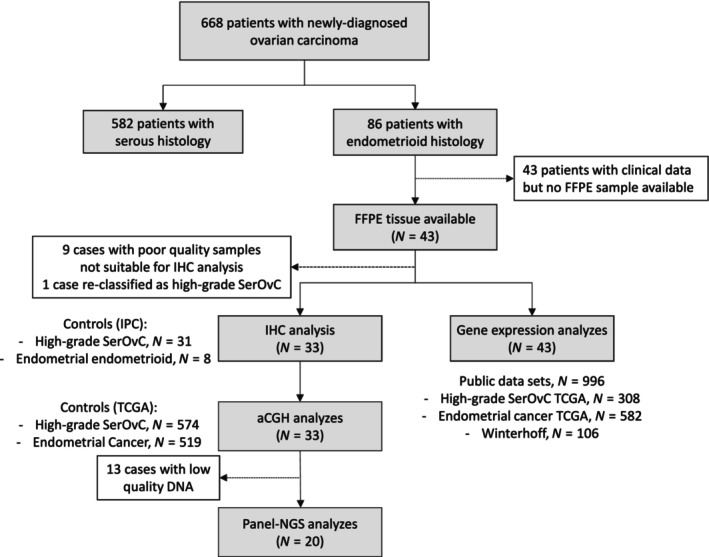
Study flow‐chart. aCGH, comparative genomic hybridization analysis; FFPE, formalin‐fixed paraffin‐embedded; IHC, immunohistochemistry.

Six hundred sixty‐eight patients diagnosed with endometrioid or serous ovarian carcinoma between 2000 and 2016 and managed in one of the three participating centers were included in our preliminary clinical analysis (Fig. 1). Of them, 86 (12.9%) were diagnosed with endometrioid ovarian carcinoma (Table S4). When compared with patients with SerOvC, women with EOvC were younger (median age 55 *vs*. 61 years), were diagnosed at earlier stage (FIGO stages I–II 49% *vs*. 16%), and had lower grade disease (low grade 53% *vs*. 39%).With a median follow‐up of 55.4 months (95CI [48.4–62.4]), five‐year OS rates were 60% in the endometrioid group and 45% in the serous group (*P* = 0.001, Fig. S1A). In a multivariate analysis including age, FIGO stage, surgery results, and chemotherapy administration, the lower risk of death from EOvC compared with SerOvC was no longer significant (HR = 0.98 (95CI [0.63–1.53]); *P* = 0.983, Fig. S1B). Advanced FIGO stage and incomplete macroscopic resection were significant poor prognosis features in multivariate analysis. Similar results were observed with PFS (HR = 1.02 (95CI [0.71–1.48]); *P* = 0.908, Fig. S1C). Forty‐three patients diagnosed with EOvC had clinical data and FFPE tissue samples available and were included in pathological/molecular analyzes. Clinicopathological characteristics of this subset were similar to that of the whole EOvC population (Table 1). As little is known about the biological discrepancies between low and high‐grade EOvC, and due to the limited sample size of the FFPE EOvC population, we thought that exploring all EOvC may bring new insights about this disease and chose to not limit analyses to a single sub‐population. We used high‐grade serous ovarian carcinoma and endometrioid endometrial carcinoma from IPC as control for IHC analysis, as well as public data sets as control for copy number and gene expression analyses (Fig. 1 and Table S5).

**Table 1:** Demographics. Data are represented as *N* (%). Percentages are calculated in relation to the number of available data.

	Endometrioid ovarian carcinoma (*N* = 86)	Serous ovarian carcinoma (*N* = 582)	*P*‐value (Khi^2^ or Wilcoxon)	Endometrioid ovarian carcinoma FFPE subset (*n* = 43)
Median age [Min‐Max]	55 [20–88]	61 [18–91]	< 0.001	62 [39–88]
FIGO stage				
I‐II	37 (49)	89 (16)	< 0.001	17 (39)
III‐IV	39 (51)	475 (84)		27 (61)
Grade				
Low	24 (53)	43 (29)	0.004	25 (63)
High	21 (47)	106 (71)		15 (37)
Surgical macroscopic resection				
Complete	55 (81)	351 (72)	0.172	27 (84)
Incomplete	13 (19)	135 (28)		5 (16)
Adjuvant chemotherapy				
Yes	72 (89)	558 (96)	0.006	37 (84)
No	9 (11)	21 (4)		7 (16)

### 3.2 IHC analysis of endometrioid ovarian cancer

**Fig. 2:**
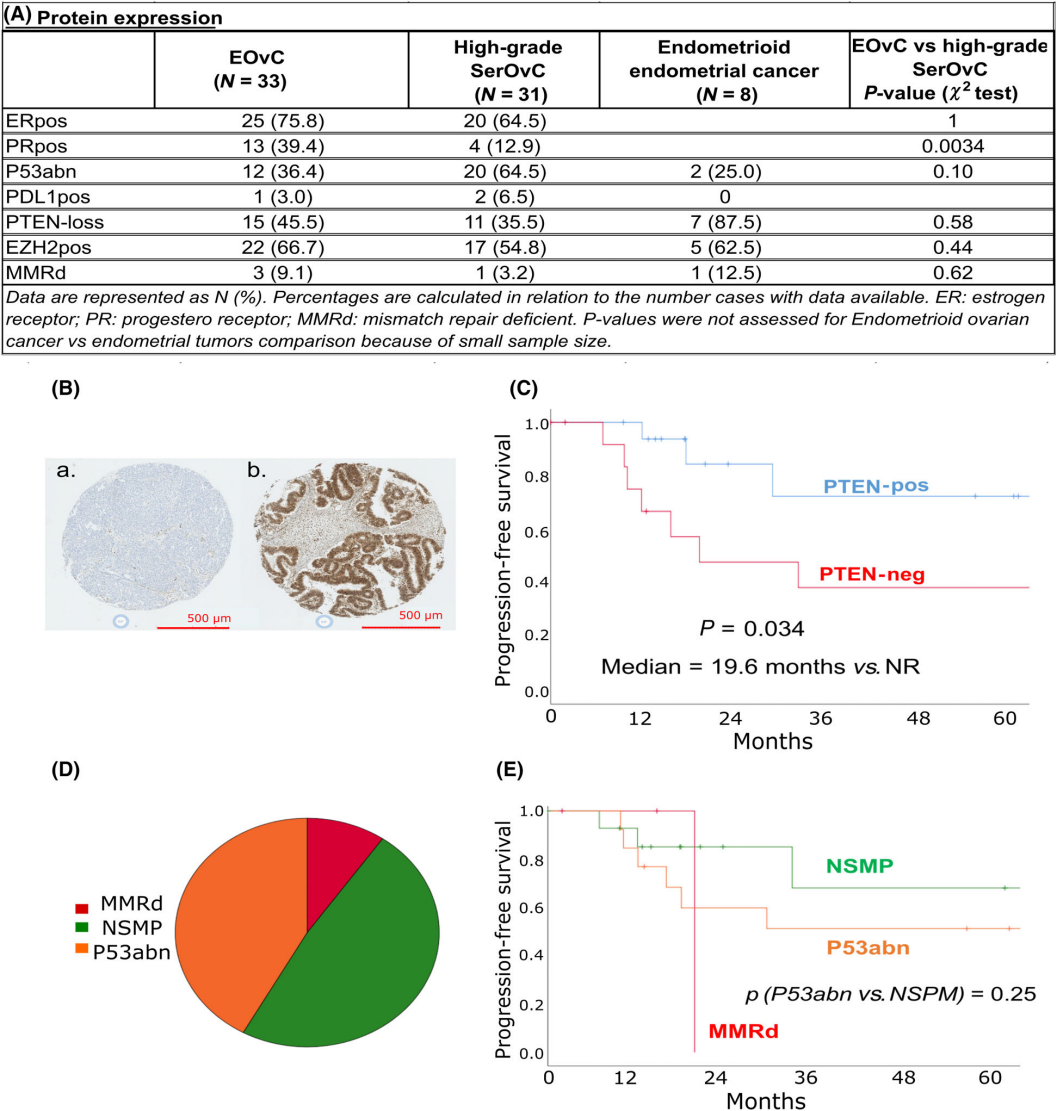
Protein expression and ProMisE classification in endometrioid ovarian cancer (EovC). (A) Protein expression levels in EOvC, high‐grade serous ovarian cancer (SerOvC), and endometrioid uterine carcinoma. (B) Examples of PTEN positive (A) and negative (B) cases identified by tissue microarray analysis (zoom ×5). (C) Kaplan–Meier curves for progression‐free survival according to PTEN expression. (D) ProMisE classification based on immunohistochemistry data. (E) Kaplan–Meier curves for progression‐free survival according to ProMisE classification. MMRd, mismatch repair deficient; NSMP, non‐specific molecular subtype. *P*‐values are evaluated using the log‐rank test.

Quality control of FFPE blocks or slides and pathological review identified nine cases not suitable for further IHC analysis. One case was classified as high‐grade serous carcinoma after pathological reviewing. Concomitantly to these 33 exploitable EOvC cases, we analyzed 31 high‐grade serous ovarian cancer as well as eight endometrioid endometrial tumors as controls (Fig. 2A).

Estrogen receptor expression was similar in EOvCs and SerOvCs (75.8% *vs*. 64.5%, *P* = 1), whereas progesterone receptor expression was higher in EOvCs (39% *vs*. 13%, *P* = 0.034). Mismatch repair deficiency (loss of MLH1/PMS2 or MSH2/MSH6) was observed in three EOvCs, one endometrioid endometrial cancer, and one SerOvC. Only very few ovarian cancer cases were PDL1 positive (one EOvC and two SerOvCs).

SerOvCs tended to display higher rates of P53 abnormalities (P53abn) profile identified by IHC (64.5% *vs*. 36.3%, *P* = 0.10). Rates of PTEN loss were similar across ovarian cancer subtypes, with 45.5% in EOvC *vs*. 35.5% in SerOvC. This rate reached 88% in our endometrial cancer controls. EZH2 protein expression was similar in all three subtypes.

#### 3.2.1 Prognostic value of protein expression in endometrioid ovarian cancer

Most of the protein markers explored by IHC were not prognostic in our endometrioid tumors set. The only protein associated with PFS was PTEN (Table S6). PTEN expression was decreased in 45% of the EOvC subset (Fig. 2B). These patients had a median PFS of 19.6 months, whereas it was not reached in the PTEN‐positive patients (Fig. 2C). This survival benefit tended to be independent of FIGO stage at diagnosis (*P* = 0.05; HR = 3.77 (95CI [0.98–14.47]), multivariate analysis including PTEN status and FIGO stage). We then explored the prognostic value of the ProMisE classification based on IHC data in the EOvC subset (*N* = 33). Nearly half (48%) of samples with data available were of non‐specific molecular profile (NSMP), 42% were P53abn, and 10% were MMRd (Fig. 2D). No significant survival difference was observed between P53abn and tumors of NSMP (Fig. 2E).

### 3.3 Copy number alterations and mutation profiles of EOvC are closer to those of endometrioid uterine cancer than to serous ovarian carcinoma

IHC analysis showed that progesterone receptor and P53 expressions were not similar between EOvC and SerOvC. We then explored DNA alterations to compare both subtypes and also to compare EOvC with endometrioid endometrial cancer.

**Fig. 3:**
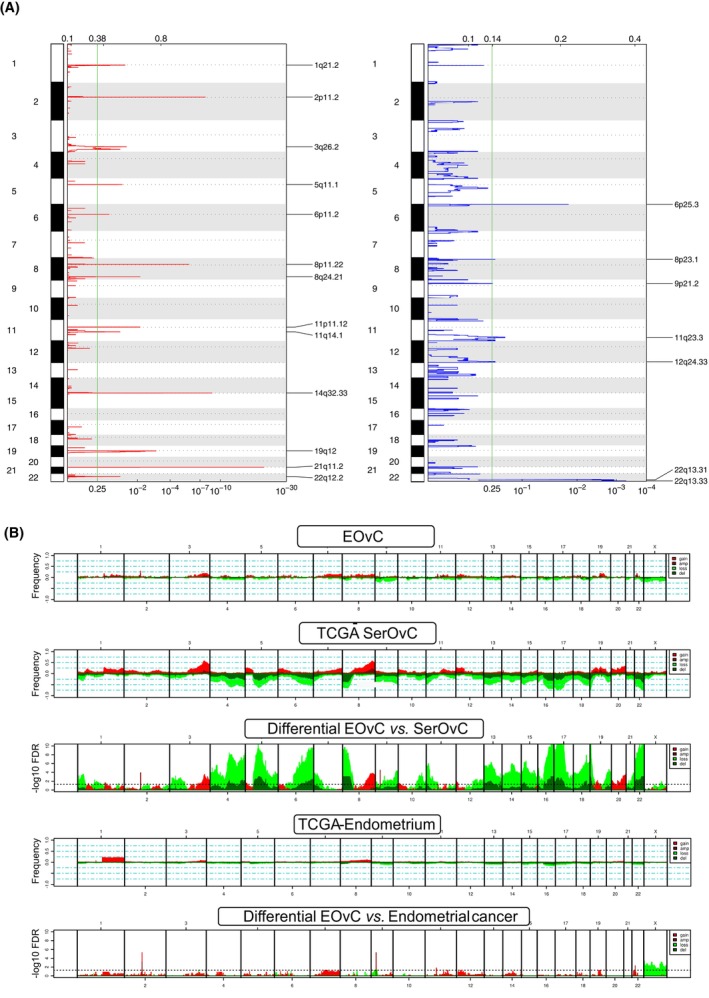
Copy number alterations in endometrioid ovarian cancer (*N* = 33). (A) Frequency plot of recurrent copy number alterations identified in endometrioid ovarian cancer (EOvC) tumors using the GISTIC algorithm. Frequencies of gains (red) and losses (blue) are plotted as a result of chromosome location. *X*‐axis: top = log–scale ratio; bottom = *q*‐values. Green lines represent the threshold for significance. (B) Supervised analysis comparing EOvC samples to high‐grade serous ovarian carcinoma and endometrial tumors from TCGA datasets. Dotted line of the bottom charts comparing EOvC to high‐grade serous ovarian cancer or endometrioid endometrial cancer: threshold of significance associated with a False Discovery Rate < 0.25.

We first performed aCGH on 33 EOvC samples. GISTIC‐2.0 analysis identified several recurrent copy number alterations (CNAs). Among others, we observed frequent gains/amplifications in 2p11.2 (*CD8A, CD8B, RGPD1*), 8q24.21 (*MYC*), 8p11.22 (*ADAM3A, ADAM5P*), 11p11 (*FOLH1*), and 3q26.2 (*MECOM*) (Fig. 3A). Deletions/losses in regions of interest were also identified: 6p25.3 (*FOXQ1*), 11q23.3 (*ATM, MMPs*), 22q13.31 (*NUP50, ATXN10, PRR5, PHF21B, PNPLA5*), and 12q24.33 (*POLE*).

Supervised analysis of CNAs identified in our EOvC cohort *versus* the TCGA endometrial and ovarian data sets showed that several regions were differentially altered (Fig. 3B and Table S7). A few regions (2p11.2, 9p21.3, 11p11.12, 22q12.2) were more frequently amplified in EOvC compared with endometrial endometrioid cancers. The EOvC *vs*. SerOvC comparison identified much more differential CNAs as SerOvC presented a higher rate of genomic instability, mainly driven by homologous recombination deficiency.

Next‐generation sequencing was performed on a subset of 20 EOvCs. Sequencing was not feasible for the other tumors due to low‐quality DNA in samples collected more than 10 years before molecular analysis. One hundred and ninety‐two somatic pathogenic alterations were identified in 192 genes (Fig. S2A and Table S8). Most of the observed alterations (63%) were missense single nucleotide variations, followed by indels (27%), nonsense mutations (7%), and splicing mutations (3%). All analyzed tumors displayed at least one mutation of interest. Most mutations were observed in homologous recombination deficiency (HRD)‐related genes (*N* = 17), PI3K pathway genes (*N* = 15), genes involved in the SWI/SNF complex (*N* = 12), and in *TP53* (*N* = 12) (Fig. S2B).

We observed *TP53* mutations in 12 tumors, homologous recombination deficiency‐associated mutations in 12 cases, PIK3/AKT pathway (*n* = 8 tumors), SWI/SNF pathway (*n* = 8), *CTNNB1* mutations (*n* = 6 tumors), and MAPK pathway mutations (*n* = 5), (Fig. S2C). Of note, *TP53* and *CTNNB1* mutations were mutually exclusive. Two patients presented a mutation in genes involved in mismatch repair: one *PMS2* and one *MSH2*. Both were classified as MMRd by IHC. No mutation in the exonuclease domain of *POLE* was identified. All these alterations have been described in endometrial cancer with similar frequencies in the TCGA dataset: *TP53* mutations in 29%, *CTNNB1* mutations in 30%, *PI3KCA* mutations in 53%, and *ARID1A* mutations in 34% [33]. The frequencies of these alterations were different in a historical cohort of high‐grade serous ovarian carcinomas: *TP53* mutations in > 95%, *CTNNB1, PIK3CA, and ARID1A* mutations in < 1% each [34].

### 3.4 EOvC is a unique entity at the transcriptomic level

Gene expression analysis was performed on 43 EOvC samples with exploitable RNA. Unsupervised analysis showed that these tumors could not be classified using *Tothill's k‐means and CLOVAR (Classification of Ovarian Cancer)* gene expression classifications that have been validated for serous tumors [50, 51], (Fig. S3A–C). Despite these classifications having a validated prognostic value in SerOvC they were not predictive of PFS in EOvC (Fig. S3D). Unsupervised classification led to the identification of two main clusters and seemed to be mainly driven by the expression of genes involved in the cell cycle and DNA repair (Fig. S3E). However, PFS was similar in both hierarchical clustering subgroups (*P* = 0.45, log‐rank test; Fig. S3F).

**Fig. 4:**
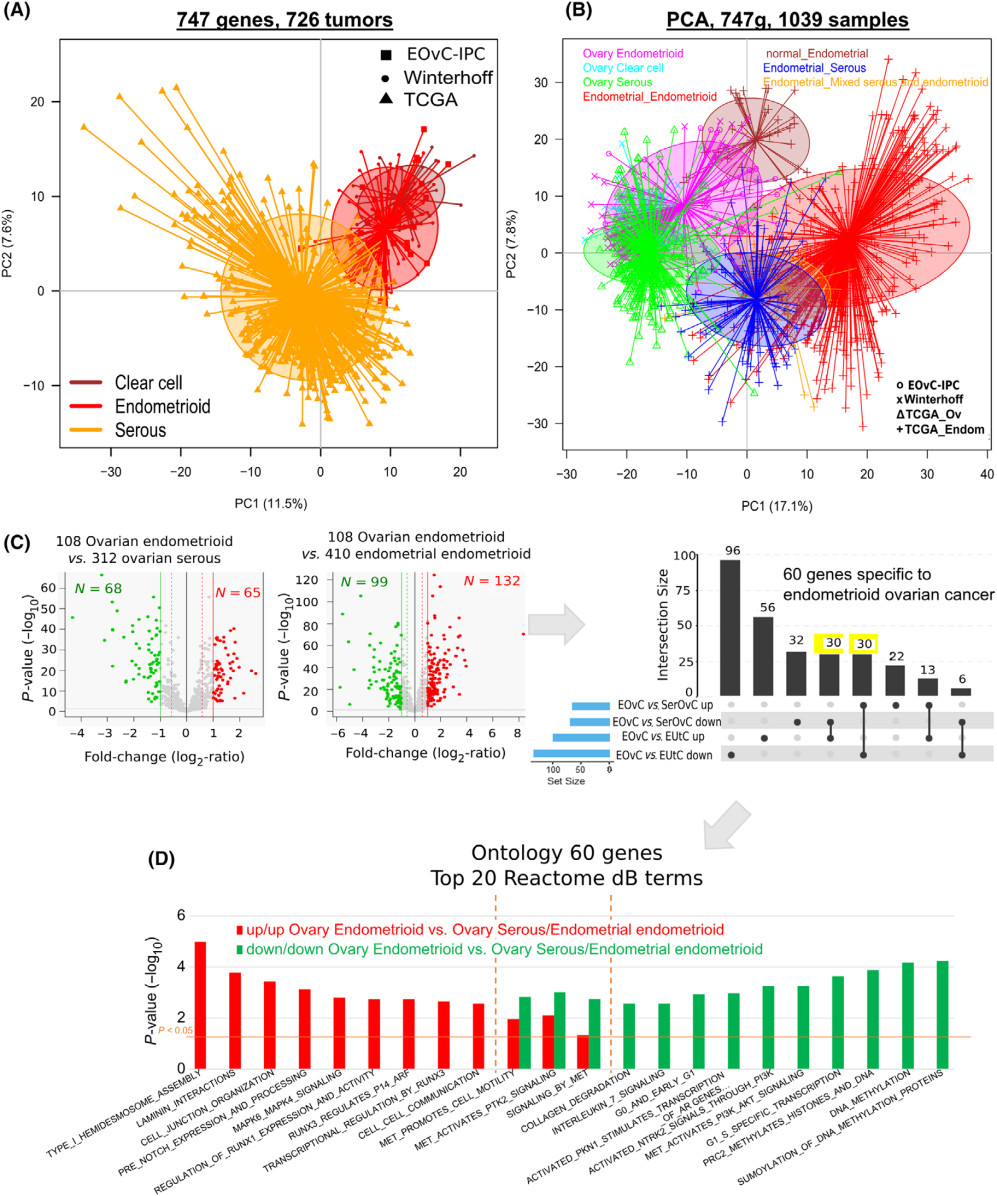
Comparison of transcriptomic profiles of endometrioid ovarian cancer (EOvC), clear cell ovarian cancer, and serous ovarian cancer (SerOvC). (A) Principal component analysis of ovarian cancers on 747 genes showing that the centroid of the EOvC set is closer to ovarian clear cell carcinoma than to serous ovarian cancer. (B) Principal component analysis including ovarian and endometrial cancer subtypes on 747 genes showing that centroids of ovarian cancer subtypes do not overlap with endometrial tumors and that EOvC are distinct from endometrioid endometrial cancer. (C) Volcano plot of differential mRNA expression between EOvC vs. SerOvC and endometrioid endometrial cancer identified 60 genes (highlighted in yellow) specifically expressed in EOvC (moderated *t*‐test: *P* < 5%, *q* < 10% & *|FC|* > 1.5×). (D) Ontology analysis of these 60 genes using the Reactome database [46, 47, 48, 50, 51].

As serous ovarian cancer classifications were not able to classify EOvC, we explored if EOvC transcriptomic profile could be compared with that of ovarian clear cell cancer. We chose clear cell tumors as they may display a similar origin with EOvC with frequent history of endometriosis in both diseases [52]. Principal component analysis including EOvC, SerOvC, and a set of clear cell OvC [46] showed that clear cell and endometrioid OvC had close gene expression profiles and can be differentiated from SerOvC (Fig. 4A).

Incorporating gene expression data of endometrial cancers from TCGA to that of ovarian tumors confirmed these results. Ovarian cancer subtypes are closer to each other than to endometrial cancer. Moreover, EOvC and endometrioid endometrial cancers did not cluster together, confirming that EOvC is a specific disease (Fig. *4*B). Supervised analyses of EOvC compared with SerOvC and of EOvC compared with endometrial endometrioid tumors identified 60 genes with altered expression specific to EOvC (Fig. *4*C, Table S9). Ontology analysis revealed that genes involved in DNA methylation and PI3K pathways were downregulated in EOvC. Genes associated with cell junction, cell‐to‐cell communication, and transcription regulation through RUNX genes were overexpressed in EOvC (Fig. *4*D).

## 4 Discussion

This multicenter retrospective study is the first study describing a pathological and multi‐omics analysis of endometrioid ovarian cancer, a rare tumor type. It shows that EOvC display specific biological features with a IHC profile similar to that of endometrial endometrioid cases, but with a gene expression profile close to ovarian clear cell carcinoma. Moreover, we identified PTEN protein expression as a potential prognostic marker in endometrioid ovarian cancer.

Retrospective analysis of clinical records of more than 600 patients showed that EOvC patients have a better outcome than serous ovarian cancer, with a 15% improvement (60% *vs*. 45%) of 5y‐OS. However, we observed that this difference is driven by the earlier stage at diagnosis with half of EOvC diagnosed at stage I‐II *vs*. < 20% in serious cases. Early diagnosis of EOvC has already been described in a large French national survey including more than 3000 serous or endometrioid ovarian cancers [5]. Five‐year OS was 81% in EOvC *vs*. 55% in SerOvC, close to what we observed in this study. The endometrioid subtype was an independent prognostic feature, even though patients with EOvC were more likely to be diagnosed at younger ages and earlier stages. Similar results were observed in another large American study based on SEER data [53]. One hypothesis for early diagnosis was that Lynch Syndrome is more frequent in EOvC than in SerOvC; and patients with a family history of Lynch Syndrome may benefit from iterative clinical and radiological assessments. Mismatch repair deficiency is indeed more frequent in endometrioid tumors than in serous ones [54, 55]. Using tissue micro‐array, we also identified some MMRd tumors (three of 33) in EOvC *vs*. none of the 30 serous controls. Regarding recent positive results of chemotherapy/immunotherapy combinations in MMRd endometrial carcinoma [22, 23, 56], these patients may be good candidate for exploring immune checkpoint inhibitors in ovarian cancer.

*TP53* mutations are observed in more than 95% of SerOvC [34]. TMA analysis identified 42% EOvC with a P53abn profile, close to what is known in endometrial cancer of various pathological subtypes [33]. Extrapolation of the ProMisE (Proactive Molecular Risk Classifier for Endometrial Cancer) to this EOvC data set based on IHC data identified 48% of NSMP cases, 42% of P53abn, and 10% of MMRd cases. This was close to the ratio described in the seminal paper of the Cancer Genome Atlas focused on endometrial cancer [33]. DNA sequencing of some of these tumors did not identify any *POLE* mutation, whereas it represents 5% of early endometrial cancer.

The PI3K/AKT pathway is frequently altered in EOvC [9]. We identified PTEN loss of expression as a potential prognostic marker in this population. Half of our set displayed PTEN loss which is a higher frequency than in SerOvC [34, 57, 58]. Patients with EOvC harboring PTEN‐loss have a poorer PFS than PTEN‐positive cases (median of 19.4 months vs not reached, *P* = 0.03). Similar observations have been made for endometrial cancer of non‐specific molecular profile [59]. PTEN is also a potential prognostic marker in SerOvC and may be correlated with lower efficacy of PARP inhibitors [57]. The prognostic impact of PTEN expression may redefine decisions on systemic treatment administration after initial debulking surgery, notably in early‐stage EOvC.

Several ovarian cancer gene expression profiling studies have been published and have proposed various gene expression classifications of high‐grade serous ovarian cancer [50, 51]. Transcriptomic subgroups defined with these classifiers were not identified in EOvC tumors from our set. As previously suggested by others, EOvC tumors display a specific mRNA expression profile [60], that may be closer to clear cell carcinoma, another rare ovarian cancer subtype derived from endometriosis lesions [46, 61]. This may lead to the development of clinical trials recruiting patients presenting with one of these two tumor types as these prospective studies are difficult to complete in each rare pathological subtype considered separately.

Our results have to be taken with caution because of some limitations. First, the small sample size of tumors available for molecular analyses has limited our capacity to explore NGS data deeply. Moreover, it has reduced our power to explore the prognostic value of numerous molecular markers. This may explain why we did not observe a significant prognostic value of the ProMisE classification in our EOvC subset, whereas it has been suggested by others [62]. The small sample size as well as the low number of events in the survival analysis according to PTEN expression also limited our capacity to identify molecular prognostic features in this population and should be confirmed in larger datasets. Moreover, it is to our knowledge, the first EOvC data set including IHC, CNA, and mRNA data. We thus were not able to confirm our results in an external validation set. Another gene expression data set of small size was available [46]. However, the small size of both data sets (~ 100 patients total) did not allow for splitting this population into training and validation sets that would have been necessary for further survival analysis. Even though it allows transcriptomic analysis on FFPE samples, the Nanostring nCounter® technology only limits this exploration to hundreds of genes, representing < 5% of the whole exome. This precluded to deeply explore whole transcriptome‐based gene expression signatures associated with response to platinum, PARP inhibitors, or immune checkpoint inhibitors. Then, our choice to perform IHC analysis using TMA may have reduced the possibility to explore the tumor microenvironment and may have led to ignoring tumor heterogeneity.

## 5 Conclusion

We have performed a comprehensive analysis of a set of endometrioid ovarian cancer suggesting that it represents a specific clinical, pathological, and molecular entity. Specific clinical prospective programs with larger sample sizes are warranted to improve our knowledge of these tumors and to develop specific clinical management.

## Conflict of interest

The authors declare no conflict of interest.

## Authors contributions

Design of the study: AdN, RS. Molecular and statistical analyzes: PF, AG, JA. Patients monitoring and data collection: RS, EK, FC, MP, EC, MAC, EL, GE, FB. Data interpretation: AdN, EM, FB, RS. Original draft: AdN and RS. Validation of the final draft for submission: all authors.

### Peer review

The peer review history for this article is available at https://www.webofscience.com/api/gateway/wos/peer‐review/10.1002/1878‐0261.13679.

## Supporting information


**Fig. S1.** Survival in endometrioid and serous ovarian carcinoma.
**Fig. S2.** NGS data.
**Fig. S3.** Transcriptomic unsupervised analysis.
**Table S1.** List of antibodies used for IHC analyses.
**Table S2.** NGS panel.
**Table S3.** List of 29 genes added to the Cancer Pathway panel for transcriptomic analysis.
**Table S4.** Clinical features of endometrioid and serous ovarian carcinoma included in the preliminary clinical analysis.
**Table S5.** Control sets for mRNA and copy number alterations analyses.
**Table S6.** Cox univariate analysis of progression‐free survival including IHC markers (N = 30).
**Table S7.** Copy number alterations supervised analysis of EOvC versus endometrioid endometrial cancer (A) and serous ovarian cancer (B).
**Table S8.** Deleterious DNA mutations identified by NGS (N = 20).
**Table S9.** List of the 60 genes identified as differentially expressed in endometrioid ovarian cancer vs. serous ovarian cancer and endometrial endometrioid cancer.

## Data Availability

All genomic and transcriptomic data analyzed in this study are available in supplementary materials.
