# Supplementary material for: Endometrioid ovarian carcinoma landscape: pathological and molecular characterization
Source: Mol Oncol. 2024 Jun 25;18(10):2586–600. doi: 10.1002/1878-0261.13679 (PMC11459045; doi:10.1002/1878-0261.13679)
Supplement: Supplementary file 1 — Fig. S1. Survival in endometrioid and serous ovarian carcinoma. Fig. S2. NGS data. Fig. S3. Transcriptomic unsupervised analysis. Table S1. List of antibodies used for IHC analyses. Table S2. NGS panel. Table S3. List of 29 genes added to the Cancer Pathway panel for transcriptomic analysis. Table S4. Clinical features of endometrioid and serous ovarian carcinoma included in the preliminary clinical analysis. Table S5. Control sets for mRNA and copy number alterations analyses. Table S6. Cox univariate analysis of progression‐free survival including IHC markers (N = 30). Table S7. Copy number alterations supervised analysis of EOvC versus endometrioid endometrial cancer (A) and serous ovarian cancer (B). Table S8. Deleterious DNA mutations identified by NGS (N = 20). Table S9. List of the 60 genes identified as differentially expressed in endometrioid ovarian cancer vs. serous ovarian cancer and endometrial endometrioid cancer. [file MOL2-18-2586-s001.zip › mol213679-sup-0013-Supinfo.pdf]

Endometrioid ovarian carcinoma landscape: pathological and molecular characterization

de Nonneville *et al.*

Supporting Information section

Supplementary Table 1. List of antibodies used for IHC analyses.

Supplementary Table 2. NGS panel

Supplementary Table 3. List of 29 genes added to the Cancer Pathway panel for transcriptomic analysis.

Supplementary Table 4. Clinical features of endometrioid and serous ovarian carcinoma included in the preliminary clinical analysis.

Supplementary Table 5. Control sets for mRNA and copy number alterations analyses.

Supplementary Table 6. Cox univariate analysis of progression-free survival including IHC markers (N=30).

Supplementary Table 7. Copy number alterations supervised analysis of EOvC versus endometrioid endometrial cancer (A) and serous ovarian cancer (B)

Supplementary Table 8. Deleterious DNA mutations identified by NGS (N=20)

Supplementary Table 9. List of the 60 genes identified as differentially expressed in endometrioid ovarian cancer vs. serous ovarian cancer and endometrial endometrioid cancer.

Supplementary Figure 1. Survival in endometrioid and serous ovarian carcinoma. (A) Kaplan Meier curves for overall survival (OS), N=668. (B) Multivariate analysis for OS, N=538. (C) Multivariate analysis for progression-free survival (PFS).

Supplementary Figure 2. NGS data (N=20). A: types of mutations observed in our set. B: number of mutations observed in each pathway of interest. C: pathways involved in each patient. SNV: single nucleotide variant, HRD: homologous recombination repair, MMR: mismatch repair, MAPK: MAP kinases, Pt: patient. Mutations *involved BRCA1, BRCA2, ATM, CHEK2, RAD51, ATR, FANCA, FANCL, ATRIP, and BRIP1* for HRD; *PIK3CA, PTEN, AKT1, PIK3R1, PIK3C2G, TSC2, and EPHB1* for the PI3K pathway; *ARID1A, EZH2, SPARCA4, SMARCA5, and ATRX* for the SWI-SNF complex; *MSH2* and *PMS2* for MMRd; and *BRAF, NF1, RASA1, and MAP3K10* for the MAPK pathway.

Supplementary Figure 3. Transcriptomic unsupervised analysis. A: hierarchical clustering of 43 EOvCs with 760 genes. B: classification of these 43 tumors by the Tothill's k-means and CLOVAR (Classification of Ovarian Cancer) classifications (45,46). Tothill k-means classification: dark blue=C1, brown=C2, light blue=C3, orange=C4, red=C5. CLOVAR classification: red=differentiated, green=immunoreactive, purple=proliferative, blue=mesenchymal. C: CLOVAR classification of 500 tumors from TCGA using whole exome TCGA gene set and the 760-gene classifier from our Nanostring analysis. We observed a high concordance (87%, *p*=8.5 E-187, binomial test) between gene sets. D: prognostic value of the same classifications in EOvC. E: ontology analysis using the GO Bioportal database of genes differentially expressed between clusters I and II. F: Kaplan Meier curves for progression-free survival in clusters identified by unsupervised analysis.
