# Supplementary figures and images for: Endometrioid ovarian carcinoma landscape: pathological and molecular characterization
Source: Mol Oncol. 2024 Jun 25;18(10):2586–600. doi: 10.1002/1878-0261.13679 (PMC11459045; doi:10.1002/1878-0261.13679)

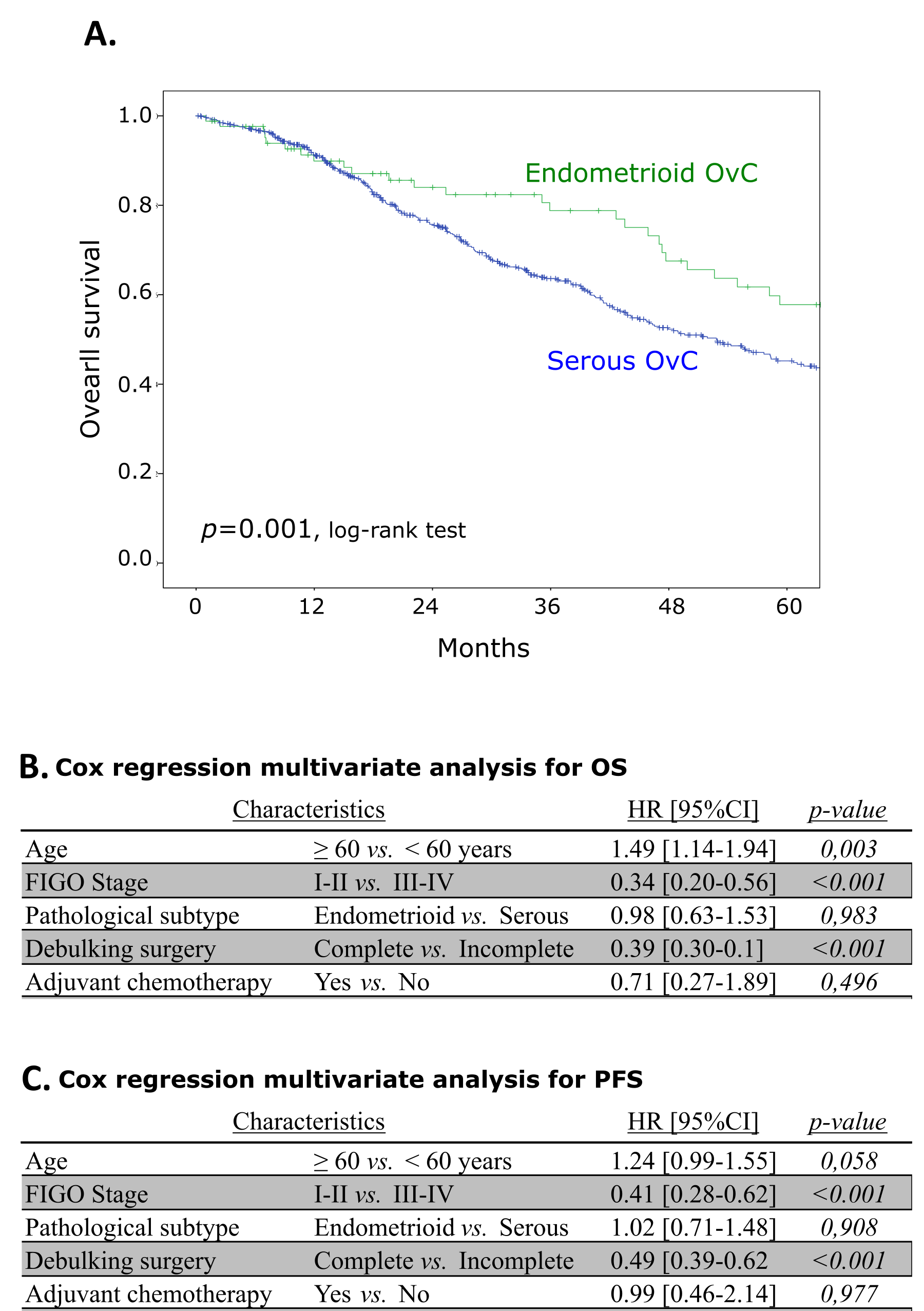

Supplement: Supplementary file 1 — Fig. S1. Survival in endometrioid and serous ovarian carcinoma. Fig. S2. NGS data. Fig. S3. Transcriptomic unsupervised analysis. Table S1. List of antibodies used for IHC analyses. Table S2. NGS panel. Table S3. List of 29 genes added to the Cancer Pathway panel for transcriptomic analysis. Table S4. Clinical features of endometrioid and serous ovarian carcinoma included in the preliminary clinical analysis. Table S5. Control sets for mRNA and copy number alterations analyses. Table S6. Cox univariate analysis of progression‐free survival including IHC markers (N = 30). Table S7. Copy number alterations supervised analysis of EOvC versus endometrioid endometrial cancer (A) and serous ovarian cancer (B). Table S8. Deleterious DNA mutations identified by NGS (N = 20). Table S9. List of the 60 genes identified as differentially expressed in endometrioid ovarian cancer vs. serous ovarian cancer and endometrial endometrioid cancer. [file MOL2-18-2586-s001.zip › mol213679-sup-0001-FigureS1.png]

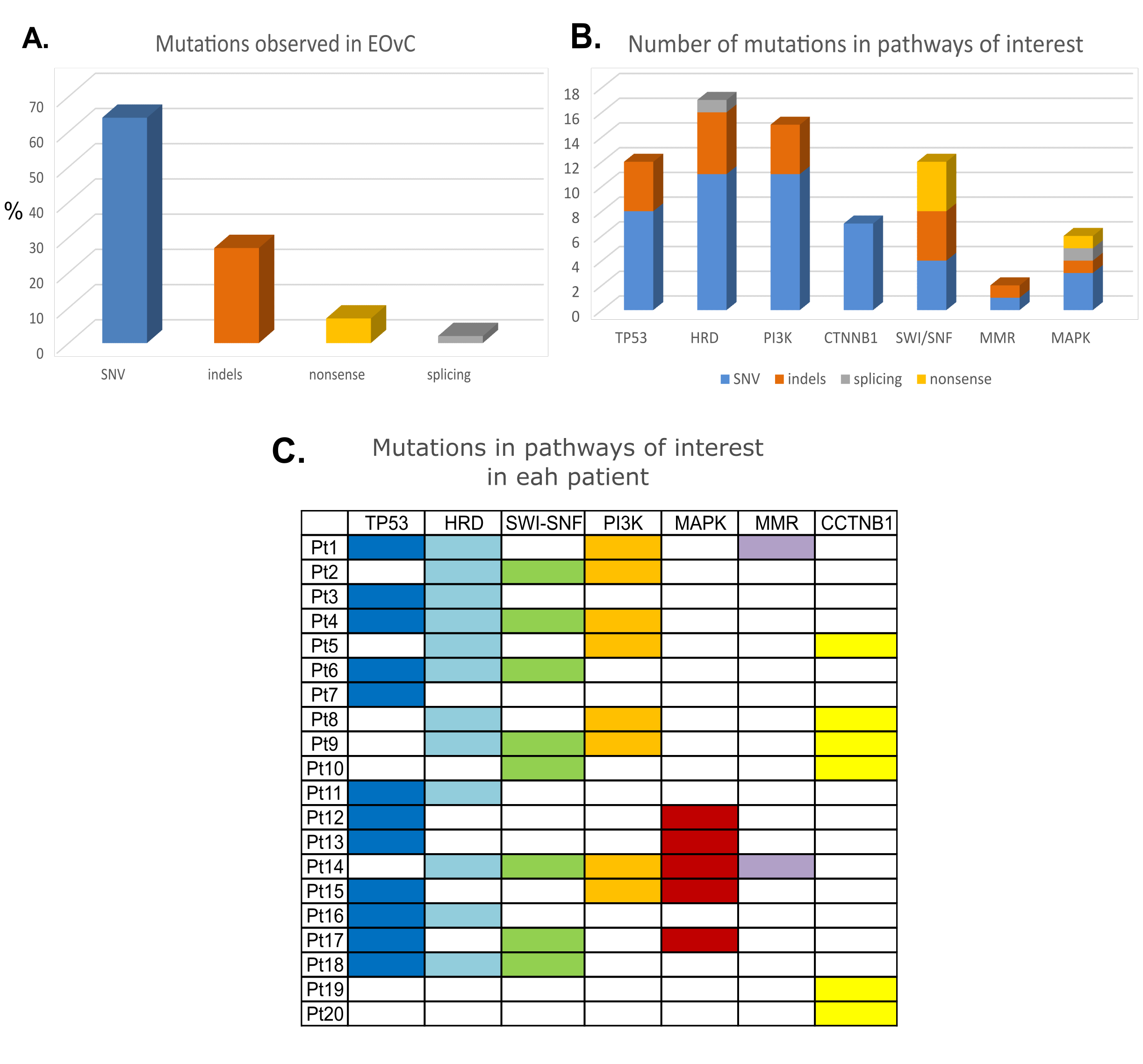

Supplement: Supplementary file 1 — Fig. S1. Survival in endometrioid and serous ovarian carcinoma. Fig. S2. NGS data. Fig. S3. Transcriptomic unsupervised analysis. Table S1. List of antibodies used for IHC analyses. Table S2. NGS panel. Table S3. List of 29 genes added to the Cancer Pathway panel for transcriptomic analysis. Table S4. Clinical features of endometrioid and serous ovarian carcinoma included in the preliminary clinical analysis. Table S5. Control sets for mRNA and copy number alterations analyses. Table S6. Cox univariate analysis of progression‐free survival including IHC markers (N = 30). Table S7. Copy number alterations supervised analysis of EOvC versus endometrioid endometrial cancer (A) and serous ovarian cancer (B). Table S8. Deleterious DNA mutations identified by NGS (N = 20). Table S9. List of the 60 genes identified as differentially expressed in endometrioid ovarian cancer vs. serous ovarian cancer and endometrial endometrioid cancer. [file MOL2-18-2586-s001.zip › mol213679-sup-0002-FigureS2.png]

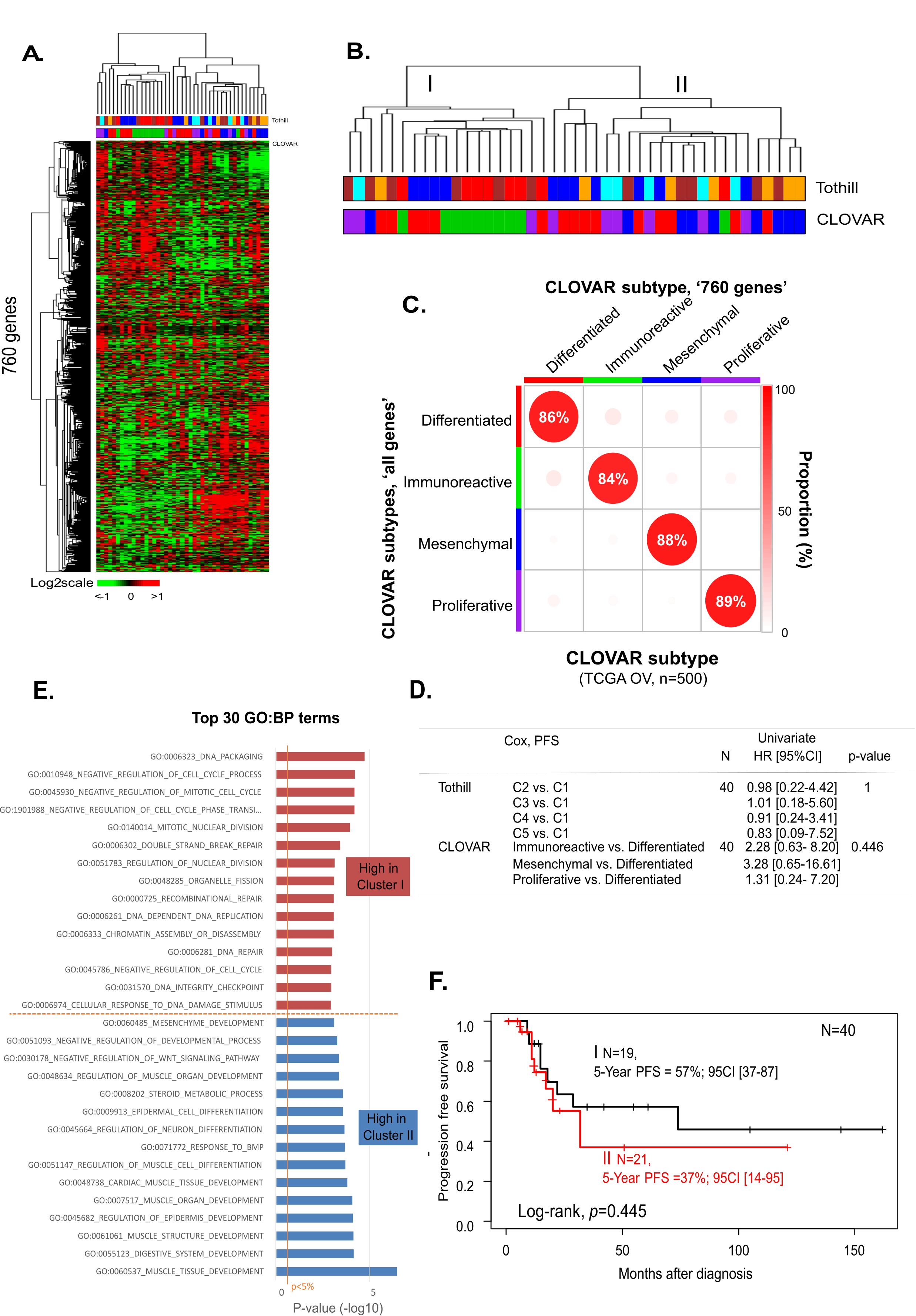

Supplement: Supplementary file 1 — Fig. S1. Survival in endometrioid and serous ovarian carcinoma. Fig. S2. NGS data. Fig. S3. Transcriptomic unsupervised analysis. Table S1. List of antibodies used for IHC analyses. Table S2. NGS panel. Table S3. List of 29 genes added to the Cancer Pathway panel for transcriptomic analysis. Table S4. Clinical features of endometrioid and serous ovarian carcinoma included in the preliminary clinical analysis. Table S5. Control sets for mRNA and copy number alterations analyses. Table S6. Cox univariate analysis of progression‐free survival including IHC markers (N = 30). Table S7. Copy number alterations supervised analysis of EOvC versus endometrioid endometrial cancer (A) and serous ovarian cancer (B). Table S8. Deleterious DNA mutations identified by NGS (N = 20). Table S9. List of the 60 genes identified as differentially expressed in endometrioid ovarian cancer vs. serous ovarian cancer and endometrial endometrioid cancer. [file MOL2-18-2586-s001.zip › mol213679-sup-0003-FigureS3.png]
